# Semi-automated curation of protein subcellular localization: a text mining-based approach to Gene Ontology (GO) Cellular Component curation

**DOI:** 10.1186/1471-2105-10-228

**Published:** 2009-07-21

**Authors:** Kimberly Van Auken, Joshua Jaffery, Juancarlos Chan, Hans-Michael Müller, Paul W Sternberg

**Affiliations:** 1 Division of Biology, California Institute of Technology, Pasadena, CA 91125, USA; 2 Howard Hughes Medical Institute and Division of Biology, California Institute of Technology, Pasadena, CA 91125, USA; 3 California Department of Transportation, San Bernardino, California 92401, USA

## Abstract

**Background:**

Manual curation of experimental data from the biomedical literature is an expensive and time-consuming endeavor. Nevertheless, most biological knowledge bases still rely heavily on manual curation for data extraction and entry. Text mining software that can semi- or fully automate information retrieval from the literature would thus provide a significant boost to manual curation efforts.

**Results:**

We employ the Textpresso category-based information retrieval and extraction system , developed by WormBase to explore how Textpresso might improve the efficiency with which we manually curate *C. elegans *proteins to the Gene Ontology's Cellular Component Ontology. Using a training set of sentences that describe results of localization experiments in the published literature, we generated three new curation task-specific categories (Cellular Components, Assay Terms, and Verbs) containing words and phrases associated with reports of experimentally determined subcellular localization. We compared the results of manual curation to that of Textpresso queries that searched the full text of articles for sentences containing terms from each of the three new categories plus the name of a previously uncurated *C. elegans *protein, and found that Textpresso searches identified curatable papers with recall and precision rates of 79.1% and 61.8%, respectively (F-score of 69.5%), when compared to manual curation. Within those documents, Textpresso identified relevant sentences with recall and precision rates of 30.3% and 80.1% (F-score of 44.0%). From returned sentences, curators were able to make 66.2% of all possible experimentally supported GO Cellular Component annotations with 97.3% precision (F-score of 78.8%). Measuring the relative efficiencies of Textpresso-based versus manual curation we find that Textpresso has the potential to increase curation efficiency by at least 8-fold, and perhaps as much as 15-fold, given differences in individual curatorial speed.

**Conclusion:**

Textpresso is an effective tool for improving the efficiency of manual, experimentally based curation. Incorporating a Textpresso-based Cellular Component curation pipeline at WormBase has allowed us to transition from strictly manual curation of this data type to a more efficient pipeline of computer-assisted validation. Continued development of curation task-specific Textpresso categories will provide an invaluable resource for genomics databases that rely heavily on manual curation.

## Background

One of the main challenges of genome projects is providing reliable functional annotations for gene products. For extensively studied model organisms, such as the nematode *C. elegans*, high quality functional annotations are largely gleaned from manual curation of experiments reported in the published literature, with additional annotations obtained from computational or comparative methods, such as protein domain analysis [1,2]. For organisms with smaller research communities, however, functional annotations may initially derive largely from computational or comparative methods which, in turn, can rely heavily upon the accuracy and completeness of model organism genome curation for providing suitable reference annotations and training sets [3-5]. Thus, the extent to which model organism databases can keep pace with annotating an ever expanding literature will potentially have an impact not only on model organism genome curation but curation of a variety of other genomes as well. Given that manual curation is unlikely to keep up with current publication rates, developing new approaches to extracting biological facts from the published literature is imperative [6].

Introduced over ten years ago, the Gene Ontology (GO) has since become the de facto resource for functional genome annotation using controlled vocabularies [7]. Divided into three distinct ontologies that describe Biological Processes, Molecular Functions, and Cellular Components, the GO is used by database curators to record key biological features of a gene product in language that is both humanly readable and computationally amenable. A key feature of the GO is that its ontologies are structured as directed acyclic graphs (DAGs) in which terms have parent-child relationships, with child terms being more specific or specialized than their respective parent(s). For example, the GO term mitochondrion is a child of intracellular membrane-bounded organelle which is, in turn, a child of intracellular organelle. Annotations made to more specialized child terms may thus be transitively made to the more general parent terms, as well: an annotation to mitochondrion is, transitively, an annotation to intracellular organelle. A second key feature of the GO is the use of evidence codes to support annotations. Evidence codes are used by curators to give an indication of the methodology that researchers use to infer facts about the genes or gene products they are studying. For example, annotation of a gene product to the Biological Process term cell division (GO:0051301) based upon a mutant phenotype that results in arrested cell division would use the Inferred from Mutant Phenotype (IMP) evidence code. Likewise, annotation of a gene product to the Cellular Component term plasma membrane (GO:0005886) based upon immunofluorescence experiments would use the Inferred from Direct Assay (IDA) evidence code. Selection of the appropriate GO evidence code thus requires information about the experiment or assay used in a publication which is often found only by reading the full text. Manual GO curation is, therefore, a labor-intensive process that if thorough can require reading and annotating the full text of hundreds, if not thousands, of publications. Thus, there is a growing need for semi- or fully-automated GO curation strategies that will help database curators rapidly and accurately identify key experimental results in the full text of research articles.

Natural language processing (NLP) applications offer a promising approach to aiding manual GO curation. Such applications use various methodologies, including classification of text within documents, to assist in associating gene products to GO terms. While some of these applications have met with considerable success in suggesting possible GO annotations, only a few take into account a real world database curation pipeline in which GO curators examine articles from a wide variety of journals and use the full text of these articles to first select a GO term and then evaluate the experimental methodology to confidently select the appropriate GO evidence code, an absolute necessity for making a GO annotation [8-16]. Thus, there is a need for text mining tools that can accurately mimic the manual curation process and thus be reliably incorporated into a database curation pipeline.

Here, we describe our strategy for using natural language processing, in particular the Textpresso text mining system [17], to curate experimentally determined subcellular localization of *C. elegans *proteins using the Cellular Component ontology of GO. Textpresso, an open source text mining tool, functions as both a simple search engine and a pattern-based, information extraction engine that employs categories of conceptually related words to semantically mark up the full text of papers. Examples of Textpresso categories include disease, phenotype, and regulation, which contain words and phrases such as 'Bardet-Biedl syndrome1', 'cell fate transformation', and 'downregulate', respectively. Textpresso searches may be performed using keywords and/or categories, and the results are presented as a list of sentences containing terms and phrases that match the search criteria (irrespective of their order in the sentence), numerically ranked according to the number of terms or phrases in the sentence that match the search string. At present, there are 19 implementations of Textpresso worldwide, including Textpresso for Neuroscience [18], budding yeast, *Drosophila*, *Arabidopsis*, and *E. coli*, with over 100 categories, such as Brain Area and Human Disease, Fly Body Parts, and *Arabidopsis *genes, to aid in searching and fact extraction.

We focused on GO Cellular Component annotations for our studies for three main reasons that suggested to us it would serve as a potential first proof of principle for developing a Textpresso-based, semi-automated curation pipeline for a heretofore fully manual approach. The first reason is that Cellular Component annotations are generally the result of a limited number of experimental strategies, namely microscopy and subcellular fractionation, thus potentially limiting the number of terms we would need to create new curation task-specific categories. Second, because we had noted during extensive manual curation that the information for the conclusion, i.e., subcellular localization, and the type of experimental assay are often stated in the same sentence, our search strategy of finding sentences that include proteins, cellular components and words or phrases referring to the experimental approach, was likely to be successful. Third, Cellular Component annotations derived from the small-scale experiments we were aiming to curate are typically made using one GO evidence code, "Inferred from Direct Assay (IDA)", thus simplifying the annotation process. In addition, there is a need for Cellular Component curation tools that make use of the full text of research articles, as authors often fail to include the results of such experiments in the abstracts of their papers. To illustrate, for a random sampling of 27 *C. elegans *proteins (see Results) only 28.4% of possible GO Cellular Component annotations could be made solely from PubMed abstracts compared to full text, with only 17.9% of those annotations as specific as annotations made from full text.

To investigate the potential usefulness of Textpresso for GO Cellular Component curation, we constructed three new Textpresso categories, termed Cellular Components, Assay Terms, and Verbs, containing terms and phrases found in a gold-standard set of sentences describing experimentally determined subcellular localization. To assess the performance of the new categories, we used them to annotate previously uncurated *C. elegans *proteins and found that by using Textpresso we were able to make 66.2% of all possible Cellular Component annotations with 97.3% accuracy. Further, by comparing the relative efficiencies of manual versus Textpresso-based curation, we find that Textpresso-based curation has the potential to improve curation efficiency at least eight-fold, and possibly as much as 15-fold, depending upon the individual curator. By incorporating a Textpresso-based Cellular Component curation pipeline into WormBase, we have moved from fully manual curation to computer-assisted validation for this data type.

## Results and discussion

### Development of New Textpresso Categories

**Figure 1:**
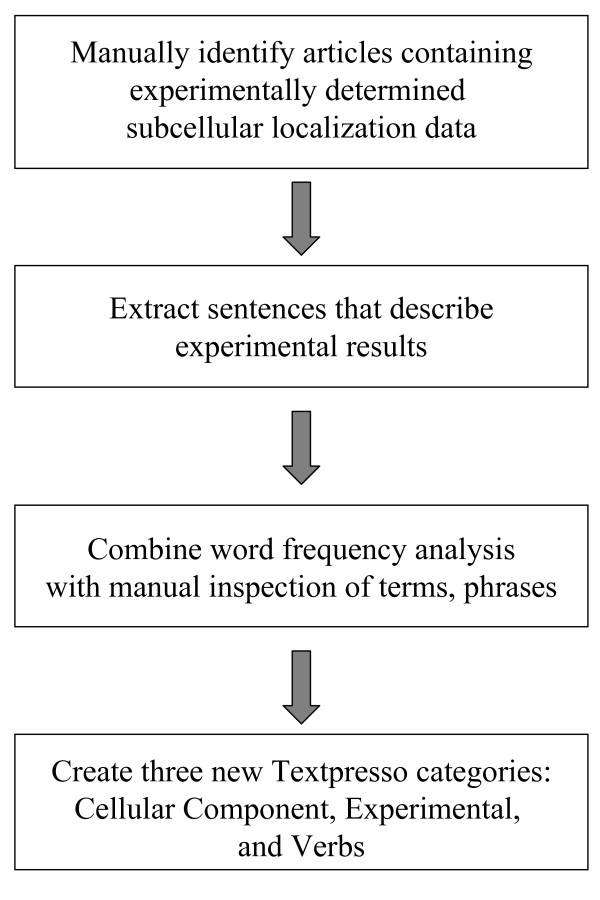
**Textpresso category development for Cellular Component curation**. Curators identified true positive sentences from a training set and used word frequency analysis and manual inspection to identify words and phrases that were most indicative of experimentally determined subcellular localization. Three new categories, Cellular Components, Assay Terms, and Verbs, were created.

**Figure 2:**
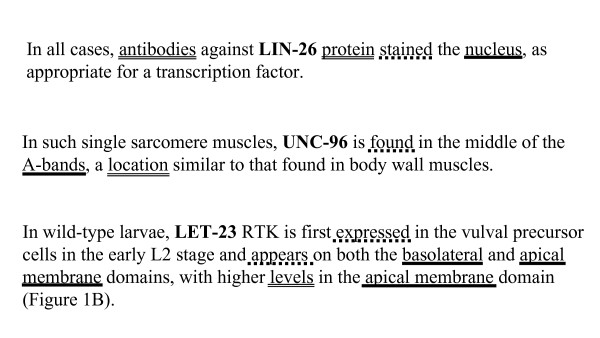
**Sample true positive sentences from the training set**. Three different sentences from the training set are shown [19-21], illustrating the types of sentences selected by curators and the individual terms selected for each of the categories. *C. elegans *proteins are shown in upper-case bold type, Cellular Components in blue, Assay Terms in red, and Verbs in green.

Our approach to developing Textpresso categories for GO Cellular Component Curation is outlined in Figure 1 and described in detail in the Methods. To identify words and phrases relevant to reports of subcellular localization experiments, we collected ~1,700 sentences from papers reporting experimentally determined subcellular localization, and then analyzed lists of words and phrases used in the sentences, as well as the frequency with which the words and phrases occur, to manually select terms that authors use to describe their experimental results (Figure 2[19-21], see Additional file 1). Words and phrases identified by our word usage and frequency analysis were then manually sorted into three categories: Cellular Components, Assay Terms, and Verbs, and included terms such as: nucleus, cell body, centrosomal; expression, antibody, throughout; and detect, exhibited, revealed, respectively (see Additional file 2).

While the division into three categories may seem somewhat arbitrary, it is semantically meaningful. Each of the three categories, along with the name of a *C. elegans *protein, captures the essential aspects of a localization experiment: a protein object, a cellular component, an action verb that reports the result, and an assay term that has a high probability of linking terms from the three other categories to an actual experimental result. Requiring a match to at least one term in each of multiple categories affords higher specificity than if all phrases were used to form just one category. In addition, employing multiple categories aids in future category development, as it makes it possible to obtain additional terms and phrases for each category by performing searches requiring matches, for example, to only three of the four categories, thereby identifying sentences that might contain new terms or phrases to potentially be included in the omitted category.

### Testing Strategy

To evaluate the performance of the three new Textpresso categories, we measured the recall and precision of searches employing the three new categories at the document, sentence, and annotation level. In essence: Did the search find the correct documents? Did the search return the correct sentences? Could a curator make the correct annotations from those sentences? In each case, we compared the results of Textpresso-based curation to a gold standard of fully manual curation.

For each test, we performed a Textpresso search for sentences that contained matches to at least one term in each of the categories, plus a match to one of 27 previously uncurated *C. elegans *proteins chosen at random. As the *C. elegans *research community strives to adhere to standard nomenclature practices (gene names consist of three- to four-letter abbreviations followed by a dash and a number, with the protein product a capitalized version of the gene name) we felt that this was a reasonable first approach to testing the effectiveness of the new categories. We also hoped that by analyzing a diverse set of proteins, we could more accurately assess the performance of the new categories on the existing corpus of *C. elegans *literature.

To evaluate the results of our test searches, we used standard metrics of information retrieval systems: recall, precision, and F-measure [22]. Recall is defined as the ratio of correct answers given by the system to the total number of possible correct answers in the text; recall thus reflects the completeness or coverage of the system. Precision, on the other hand, is defined as the ratio of the number of correct answers given by a system to the total number of answers given by the system; precision thus reflects the relevance of the search results. In addition, the F-measure, or F-score, is reported as an indication of the accuracy of the test.

### Test Results: Documents

For the 27 proteins in our test set, Textpresso searches using the protein name and the three new categories returned 55 primary research articles (51 unique articles, with two articles returned multiple times, see Additional file 3) with the maximum number of papers returned being 12 for the LAG-2 protein and the minimum being zero, for the AGR-1 and PAK-1 proteins. As determined manually, the 27 proteins in our test set were associated with 43 papers containing subcellular localization data in the WormBase bibliography. Therefore, to assess recall, we divided the number of correct papers returned by Textpresso, 34, by the total number of papers, 43, containing subcellular localization data for all of the genes in our test set as determined manually. At the document level, our recall rate is thus 79.1%. To determine the rate of precision, we divided the number of correct papers returned, 34, by the total number of papers returned, 55, to determine that the precision of our searches was 61.8%. Thus, for document retrieval we achieved an F-score of 69.5% (Table 1).

**Table 1:** Precision, recall, and F-score for Textpresso-based Cellular Component curation

**Test Set**	**Precision**	**Recall**	**F-score**
Documents	61.8%	79.1%	69.5%
Sentences	80.1%	30.3%	44.0%
Annotations	97.3%	65.7%	78.8%

### Test Results: Sentences

To determine the precision and recall rates of returned sentences, we took the following approach: each sentence in every document of our test set (51 unique papers returned by Textpresso plus nine false negative papers) was examined manually in order to select all true positive sentences. For these purposes, we defined true positives as those sentences that describe experimentally determined subcellular localization. The true positive sentence set thus includes all sentences from which a curator could make GO annotations, as well as those sentences that, while describing subcellular localization, would not automatically result in a GO annotation. For example, sentences describing a protein's subcellular localization in a wild-type background are suitable for GO annotation, while sentences describing localization in a mutant background, although often reported with similar, if not identical language, would not typically be annotated for GO. Reducing or eliminating the latter type of sentences (~15% of the total number of returned sentences in our test set) from our search results will be a focus of future tool development (see Conclusions).

By manual inspection, we identified 386 true positive sentences in the 60 articles we analyzed (51 unique articles returned by Textpresso plus nine false negative articles). Of the 386 true positive sentences, only 117 were returned by Textpresso, giving a recall rate, at the sentence level, of 30.3%. The precision rate, however, was considerably higher, at 80.1% (117 true positives/146 total sentences returned). The F-score for sentence returns was thus 44.0% (Table 1).

### Recall and Precision: GO Annotations

One of the main motivations for developing our new Textpresso categories was to improve the efficiency with which we assign GO Cellular Component annotations. Therefore, we also evaluated the recall and precision of the GO annotations made from our search returns. For the purposes of this analysis, we scored each annotation just once regardless of the number of times the information supporting that annotation was stated in the paper, as this reflects the normal curation process in which curators do not need to make a separate annotation for repeated statements of the same experimental result.

From the results of our Textpresso searches we were able to make 45 of 68 possible GO annotations made by manual curation. Thus, our annotation recall was 66.2%. Of the 45 annotations we made from Textpresso sentences, 44 were made to the exact same term as from manual annotations, with only one annotation made to a more general term that is a direct parent term of the manually chosen term in the Cellular Component ontology. Thus, the precision of annotations made from our Textpresso searches was 97.3% and the resulting F-score for GO annotations was 78.8% (Table 1).

### Search Failures: Analysis of False Negative and False Positive Sentences

To more thoroughly evaluate the results of our searches, and to improve upon the content of our first-draft categories, we undertook an analysis of the false negative and false positive sentences identified by our test searches. For false negative sentences, we focused on a subset of the sentences, namely those that, when missed, resulted in complete loss of an annotation from a paper. In theory, failure to make an annotation from Textpresso sentences could be due to several reasons including authors' use of non-standard protein nomenclature, distribution of relevant terms over several sentences as opposed to all terms being present in a single sentence, incompleteness of our categories, and sentences that reported subcellular localization results without using terms from all of our search categories. In practice, careful evaluation of our false negative sentences revealed that all of these factors, often in combination, contributed to failed searches. We thus classified each false negative sentence according to one or more reasons for which it failed to be returned in our searches (Table 2).

**Table 2:** Analysis of false negative sentences

**Reason(s) for search failure**	**Percentage of total sentences (n = 78)**
Non-standard protein nomenclature (NSN)	39.7% (n = 31)
Missing category term(s) (MT)	3.8% (n = 3)
Information spread over multiple sentences (IMS)	11.5% (n = 9)
Information expressed with <3 categories (IFC)	6.4% (n = 5)

NSN + MT	7.7% (n = 6)
NSN + IMS	3.8% (n = 3)
NSN + IFC	3.8% (n = 3)
MT + IMS	10.3% (n = 8)
MT + IFC	1.3% (n = 1)
NSN + MT + IMS	6.4% (n = 5)

Technical issues	5.1% (n = 4)

Of the 78 false negative sentences that we examined, we found that 31 of them (39.7%) were not returned solely because of authors' use of irregular or non-standard *C. elegans *nomenclature, with non-standard nomenclature a contributing factor in an additional 21.7% of false negative sentences that were missed due to use of non-standard nomenclature (NSN) plus: missing category terms (Table 2, NSN + MT); information spread out over several sentences (Table 2, NSN + IMS); information expressed using terms from fewer than the three categories our search required (Table 2, NSN + IFC); and a combination of non-standard nomenclature, missing terms, and information spread out over multiple sentences (Table 2, NSN + MT + IMS). Amongst these false negative sentences, there were several different nomenclature variations that deviated from the accepted standard. In one paper, PAK-1, the *C. elegans *ortholog of mammalian p21-activated kinase, was referred to as CePAK, while in another, experiments describing localization of MAA-1, a membrane-associated acyl-CoA binding protein, referred to localization of wtMAA-1 using the 'wt' prefix to denote a wild-type versus mutant protein. In another example, the AGR-1 protein was referred to throughout a publication by the name of its mammalian ortholog, agrin. These results indicate that even though the *C. elegans *community strives to use standardized nomenclature, reliably identifying gene and protein names in the *C. elegans *corpus will require cataloging and then incorporating protein synonyms in our searches, as well as exploring additional approaches to entity recognition (See Conclusions).

For nine sentences (11.5%), we missed the annotation because the relevant information was distributed amongst several sentences. In all of these cases, the protein product was at least one, if not the sole, piece of information missing from the sentence. Distribution of information over several sentences was also a contributing factor in an additional 20.5% of our false negatives (Table 2: NSN + IMS; MT + IMS; and NSN + MT + IMS), indicating that reliably caprturing information spread out over multiple sentences will be an important area of future investigation. In only three cases (3.8%), did we miss sentences solely due to the incompleteness of our categories. Overall, however, missing category terms were a contributing factor in an additional 25.7% of false negative sentences (Table 2: NSN + MT, MT + IMS; MT + IFC; and NSN + MT + IMS). These results suggest that whereas we can, and will, continue to improve the content of the categories, simply adding more terms and phrases to the categories will not, by itself, dramatically improve the number of annotations we can make using Textpresso, as we will still need to address the issues of use non-standard protein nomenclature, information spread out over multiple sentences, and results expressed using terms from fewer than the three categories we required.

In five sentences (6.4%), we found that although curators could have made an annotation directly from those sentences, they were not returned because the localization information was expressed using words from fewer than three categories. In most of these cases, the missing term was from the experimental category, as authors simply stated that the protein was localized to a particular cellular component, for example "...DAF-9 also appeared in the cytoplasm of the hypodermis...". Since our test searches required a match to the protein in question plus at least one term from each of the three categories, these sentences were missed. Lastly, in four cases (5.1%) we missed an annotation for technical reasons. In these cases, either the relevant information was presented in table format or the protein name was unidentifiable after the PDF-to-text conversion.

To understand the basis for the 29 false positive sentences, we again analyzed the content of each of the sentences. The results are presented in Table 3. For 13 of the 29 sentences, 44.8%, we determined that the text returned by the searches was not a single sentence but rather, due to technical issues with the PDF-to-text conversion, a run-on of one or more sentences from the text or, in some cases, the entire content of a table. In five cases (17.2%), the sentence was returned due to the inclusion of the term 'transmembrane' in our Cellular Component category. In these cases, we found that while 'transmembrane' is used to describe a protein domain it is not consistently used to describe experimentally determined subcellular localization and we have since removed it from the Cellular Component category. In the remaining 11 cases (37.9%), the false positive sentences were returned due to linguistic ambiguity associated with certain terms included in our categories. Examples include the term 'signal,' which can be used to describe a fluorescent signal, but also a cell-cell signal involved in intercellular communication, 'processes,' which can refer to cellular projections but also to biological events, such as cell division or aging, and 'soma' which can refer to a cell body apart from cellular projections or to somatic, as opposed to germline, tissue. In none of these cases, however, did the return of a false positive sentence result in an erroneous annotation.

**Table 3:** Analysis of false positive sentences

**Reason for search failure**	**Percentage of total sentences (n = 29)**
Run-on sentence or table contents	44.8% (n = 13)
Inappropriate category term	17.2% (n = 5)
Linguistic ambiguity	37.9% (n = 11)

### Evaluating Curation Efficiency: Textpresso-Based vs. Fully Manual Curation

Our primary motivation for developing Textpresso categories for Cellular Component curation was to improve the efficiency with which we make GO Cellular Component annotations. We therefore investigated the relative efficiencies of Textpresso versus manual curation by comparing the amount of time it took curators to curate subcellular localization data from a test corpus using each of the methods. Three curators manually examined one of three sets of twenty randomly selected papers (see Additional file 4) and recorded the amount of time it took to locate and extract subcellular localization information. Curators then traded paper sets and performed the same task, only this time using sentences returned by Textpresso.

**Figure 3:**
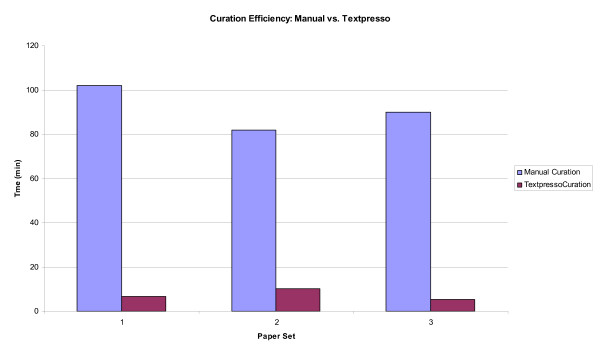
**Textpresso-based curation is more efficient than manual curation**. Three different curators recorded the amount of time it took to identify cellular component information from a set of 20 papers either read manually or searched via Textpresso using the three new cellular component categories. Textpresso-based curation results in an 8–15-fold improvement in curation efficiency depending upon the individual curator and the paper set.

The results of this curation efficiency test are presented in Figure 3. The time required for each curator to manually evaluate and record subcellular localization information from their test set of 20 papers was 102, 82, and 90 minutes each, respectively. Using Textpresso, however, the time required to make annotations was approximately 7, 10, and 6 minutes, respectively. Thus, Textpresso-based Cellular Component curation has the potential to improve curatorial efficiency by at least a factor of 8, and possibly as much as 15, given differences in individual curatorial speed. Therefore, even though our new Textpresso categories are not yet able to recover every annotation from the literature, we believe that Textpresso-based curation can still greatly improve the efficiency with which information is extracted from the literature and thus, affords a significant improvement to our GO curation pipeline.

## Conclusion

Efficient curation of biological facts from the published literature is essential for providing database users with the most complete and up-to-date information possible. Currently, the vast majority of experimental results entered into model organism databases such as WormBase are entered manually by curators who need to identify appropriate papers, read the full text, evaluate the information, and enter annotations using curation tools. Exploring ways to improve efficiency at one or more of these steps may greatly enhance the rate at which new information is culled from the literature.

We have developed a semi-automated curation strategy for annotation of *C. elegans *proteins to Gene Ontology Cellular Component terms. This approach employs the Textpresso information retrieval system which marks up the full-text of research articles using terms and phrases from conceptually related categories. Using categories constructed specifically for Cellular Component curation to annotate previously uncurated proteins, we found that we were able to retrieve 66.2% of all possible annotations in our test set with a precision of 97.3%, yielding an F-score of 78.8%. This result indicates that a Textpresso-based curation pipeline for GO Cellular Component curation is capable of retrieving a significant proportion of subcellular localization data from the literature with high precision, thus making it feasible to incorporate such an approach into our GO curation pipeline.

Indeed, having created and tested these categories for GO Cellular Component curation, we have now implemented a curation pipeline whereby each week papers newly entered into our curation database are automatically searched using *C. elegans *protein names and the three categories. Resulting sentences are displayed in a web-based curation form that allows users to enter GO annotations directly into the curation database using the associated publications as evidence (see Additional file 5). When an annotation is entered into the database, the Cellular Component category term found in the returned sentence and the Gene Ontology term selected for annotation are recorded in a relationship index, so that for all future occurrences of a given Cellular Component category term, a list of suggested Gene Ontology terms, based upon previous curation, is available to curators. Thus, Cellular Component curation at WormBase is moving from manual extraction and annotation to computer-assisted validation. Combined with the improved efficiency that Textpresso-based curation affords, this new curation pipeline has allowed us to keep current with Cellular Component annotations using substantially less curator time. As this system affords considerable time savings even for a relatively small corpus (the *C. elegans *literature is about 800 papers per year), it may well provide major time savings for larger literatures.

Continued improvements to both Textpresso and our curation pipeline may further improve curation efficiency. A thorough analysis of the false negative and false positive sentences highlighted some general considerations for future development. The false negative sentences in our test set were missed largely due to use of non-standard protein nomenclature and to distribution of relevant information over several sentences. To address the issue of protein name recognition, we can catalog and include all available synonyms in our searches, as well as make use of true positive sentences from our training and test sets (over 2,000 sentences) to employ machine-learning approaches to entity recognition [23-25]. Likewise, we can use cases where relevant information is distributed over multiple sentences to begin to develop approaches to extracting such information using Textpresso. With regard to protein names and entity recognition, however, the importance of standardized nomenclature and consistent use of this nomenclature in the published literature cannot be overstated.

We will also investigate the usefulness of search returns in which one or more categories are systematically eliminated to determine the extent to which such searches can help identify new gene product names as well as potentially new terms for the three Cellular Component categories. A benefit, but also potential drawback, of our approach is that it relied primarily on expert curator judgement for choosing terms and assigning them to appropriate categories. While this approach ensured that our category terms were highly relevant for experimentally determined localization there is, nonetheless, potential for bias in term selection. Although we note that few GO annotations were missed solely due to missing category terms, we recognize that future category development would likely benefit from incorporating additional statistical methods as well as efficient, systematic testing that would allow curators to readily compare, for example, the results of searches using a curated set of verbs versus a set comprised of normalized terms that includes all possible tenses for a given verb. Category construction will undoubtedly be an iterative process and we anticipate that further category development and testing will continually improve our results.

Although the number of false positive documents and sentences in our test set was not a hindrance to curation, improvements to further reduce the false positive rate may be possible. Textpresso searches rely upon accurate conversion of PDF documents to text, but long lists of information in tables or inadvertent run-on sentences can lead to false positive returns. Prospects for improving these technical issues include obtaining papers in XML format and improving PDF-to-text conversion.

In addition, in analyzing the false positive sentences, we found that nearly 60% of these sentences came from outside the Results section of the papers, i.e. the Introduction, Discussion, or Materials and Methods. If curators could limit searches to the Results section of relevant papers, the false positive rate could be greatly reduced. We have recently implemented a section-tagging feature in Textpresso and find that it is helpful not only for reducing the number of false positives, but also for helping curators confirm that a given sentence returned by Textpresso indeed derives from experimental data (A. Rangarajan, R. Fang, H.-M. Müller, and P. W. Sternberg, unpublished data). Lastly, we will investigate the use of exclusion terms in our searches to help lower the rate of returned sentences that describe localization in a mutant, as opposed to wild-type, background.

### Beyond *C. elegans *and Cellular Components

Recently, the Gene Ontology Consortium has undertaken a Reference Genome Project in which the genomes of twelve representative, or reference, organisms are being comprehensively annotated [26]. One of the primary motivations for undertaking the Reference Genome project was to facilitate annotation of newly sequenced genomes using experimentally supported GO annotations from the reference genomes. Tools such as Textpresso that improve efficiency of manual GO curation may thus benefit not only the reference genome communities, but additional communities that rely on reference or model organism curation for reliable functional annotation of their genomes. As Cellular Component curation is just one aspect of comprehensive genome annotation, we plan to extend the use of Textpresso-based curation to other data types, including Molecular Functions such as enzymatic activity and nucleic acid binding, to continue to improve the depth and efficiency with which genomes are annotated.

## Methods

### Textpresso for Cellular Component Curation Implementation

The Textpresso text mining system used for these studies, Textpresso for CCC, was built essentially as for other Textpresso applications. The *C. elegans *literature corpus we used derives from the WormBase bibliography in which papers identified by PubMed searches performed using the keyword '*elegans*' are manually screened to identify those papers that discuss *C elegans *and thus should be included in WormBase. Selected papers are then manually downloaded from journals' web sites in PDF format and the content of each paper converted to plain text using PDF-to-text conversion software. The converted text is subsequently processed to parse the text into individual sentences, tokenize words, and identify parts of speech. Lastly, words and phrases in the text are marked up using XML and the lexicon of existing Textpresso categories including, for our purposes, the three new categories for Cellular Component curation. An example of a sentence, before and after Textpresso mark up, is shown below:

We examined embryos at stages between late prophase and metaphase of the first mitotic division, when all wild-type embryos have foci of GFP:SAS-4 associated with both sperm centrioles (Figure 1C; n = 41). [27]

</sentence id=s'106'>We <verbs> examined </verbs> <life_stages_celegans> embryos </life_stages_celegans> <spatial_relation> at </spatial_relation> <assay_terms> stages </assay_terms> between <time_relation> late </time_relation> <biological_process> prophase </biological_process> and <biological_process> metaphase </biological_process> of the <localization> first </localization> <biological_process> mitotic </biological_process> <biological_process> division </biological_process>, when all <phenotype_celegans> wild-type </phenotype_celegans> <life_stages_celegans> embryos </life_stages_celegans> have <assay_terms> foci </assay_terms> of <assay_terms> GFP </assay_terms>: <protein_celegans> SAS-4 </protein_celegans> <verbs> associated </verbs> with <consort> both </consort> <anatomy_celegans> sperm </anatomy_celegans> <cellular_components> centrioles </cellular_components>

### Ontology Development for Cellular Component Curation

To identify words and phrases to include in the new Textpresso categories, we manually read the full text of 241 papers published in 46 different journals that reported localization experiments for 116 different *C. elegans *proteins. Selection of papers for this training set was essentially random and, while broadly representative of the literature, was unlikely to cover all possible subcellular localization scenarios. For identifying key words and phrases to include in our new categories, we used a web-based curation form in which each paper in our training set was broken down into individual sentences with a check box next to each sentence that allowed curators to select those sentences that described results of subcellular localization experiments. The resulting 1,713 'gold-standard' sentences chosen by curators were then saved as a text file for further processing (see Additional file 1).

From the text file of training sentences, we then calculated the frequency at which all one-, two-, three-, and four-word phrases appeared, excluding stop words such as determiners ("the") and prepositions ("for") from the one-word frequency analysis. By examining the list of one- through four-word phrases we were able to identify, using our knowledge of cell biology, individual words, such as 'immunofluorescence' or 'localized,' as well as phrases, such as 'endoplasmic reticulum' or 'plasma membrane,' that were associated with descriptions of subcellular localization in our training set. Although the frequency with which an individual word or phrase appeared in the training set was not necessarily the deciding factor for inclusion into our categories, we nonetheless used the frequency analysis to try to lessen our bias against words and phrases that may not have been immediately obvious as good candidates at the outset.

For each of the selected words and phrases, we then manually determined to which of the three categories, Cellular Components, Assay Terms, and Verbs, it should be assigned. Upon completion of term assignment, the resulting categories contained 160, 65, and 69 words or phrases, respectively (see Additional file 2). Words and phrases in each category were subsequently processed to include variants, such as upper and lower case, when appropriate. We note that some Cellular Component terms, such as EEs (an abbreviation for early endosomes), do not appear in the literature in lower case form and thus are represented solely in their upper case form in our category. In processing the selected terms, we did not perform stemming (reducing terms to their base form) or expand our verb list to include all verb tenses as we wished to initially create and test categories using only language that curators felt most closely reflected the stereotypical way that researchers express experimental results. To illustrate, the categories contain words such as 'broadly', 'largely', and 'seen'. If stemmed versions of terms such as these were included, search specificity would likely have suffered.

### Testing: New Category Evaluation

To determine the effectiveness of the new categories, we determined the precision, recall and F-measure for each of three tasks: 1) identification of papers containing subcellular localization experiments; 2) identification of individual sentences describing subcellular localization experiments within papers; and 3) annotation of *C. elegans *proteins to GO Cellular Component terms using Textpresso-identified sentences. For each task, the performance of the Textpresso categories was compared to that of a curator manually curating each of the papers in the test set. Test set papers were restricted solely to research articles since reviews and meeting abstracts, while included in the Textpresso corpus, are not used for GO curation. Precision, recall, and F-measure were defined as shown below.







For the first task, paper identification, we manually determined, using the WormBase bibliography, the set of papers that reported subcellular localization experiments for each of the proteins in our test set. We then compared manually identified papers to the papers returned by Textpresso scoring as true positives those papers for which one or more sentences describing localization data were returned by Textpresso. For the second task, sentence identification, we manually examined every sentence in each of the 60 test set papers to determine if it described subcellular localization and then compared the results of our manual classification to the results of Textpresso searches. For the third task, we compared both the total number and the similarity of GO annotations made using each method. Since curators typically create only one annotation per report of subcellular localization per paper, for scoring purposes we counted each cellular component annotation we made only once even if the relevant information was stated multiple times within the same paper.

### Testing: Curation Efficiency

To assess the relative efficiency of manual versus Textpresso-based curation, we selected 60 papers at random, divided them into sets of 20 papers, and assigned each set to one of three curators. The papers we chose were all primary research articles, but were otherwise not filtered for journal, proteins, or types of experiments. Each curator was assigned the task of manually scanning the papers in their set to identify, whenever reported, *C. elegans *proteins and their subcellular localization. For each paper, curators recorded the amount of time it took to identify the relevant information. To compare this manual curation rate to that of Textpresso-based curation, curators then traded sets of papers and recorded the amount of time it took to review corresponding sentences returned by Textpresso, using our test criteria that required each sentence to contain a *C. elegans *protein plus a match to at least one term in each of our three categories, and identify the same information: a *C. elegans *protein plus its reported subcellular localization.

### Literature Corpus

PubMed identifiers (PMIDs) for papers used in our training set, test set, and curation efficiency studies are available as Additional files 1, 3, and 4.

## Authors' contributions

KV and JJ identified training set sentences and developed the categories. KV performed and analyzed the recall and precision tests. KV, JJ, and PWS performed the curation efficiency tests. JC developed all curation tools for the project. HMM developed and maintained the Textpresso for Cellular Component Curation site. KV wrote the paper with valuable discussions and critical contributions at all stages of the project from HMM and PWS.

## Supplementary Material

Additional file 1 **Training set corpus and true positive sentences for Cellular Component category development**. This file contains sentences selected as true positives (the gold- standard set), for Cellular Component curation category development. Sentences are listed according to the unique PubMed identifier of the publication and the sentence number as assigned during the Textpresso PDF-to-text conversion. Click here for file

Additional file 2 **Category terms**. This file lists terms included in the first draft of the three new Textpresso categories used for GO Cellular Component curation: Cellular Components, Assay Terms, and Verbs. Click here for file

Additional file 3 **Annotation test corpus**. This file contains a list of PudMed identifiers for papers included in the test set used to evaluate the new Textpresso categories. Click here for file

Additional file 4 **Curation efficiency test corpus**. This file contains a list of PubMed identifiers for papers included in the curation efficiency test. Click here for file

Additional file 5 **WormBase Cellular Component curation form**. This file shows a screenshot of the web-based curation form used for Textpresso-based GO curation at WormBase. The identified *C. elegans *protein is listed in the left-most box, with the component term(s) from the sentence listed in the middle box. Suggested GO annotations, based upon previous curation, are listed in the right-most box. The sentence from which the protein and component are derived are shown on the right, along with the possible actions that can be taken by a curator, including curating the information (and adding to GO), marking the information as already curated, or marking the returned sentence as 'scrambled', false positive, or not GO curatable for additional reasons, e.g. the sentence describes localization in a mutant background. Click here for file
