# Supplementary figures and images for: Semi-automated curation of protein subcellular localization: a text mining-based approach to Gene Ontology (GO) Cellular Component curation
Source: BMC Bioinformatics. 2009 Jul 21;10:228. doi: 10.1186/1471-2105-10-228 (PMC2719631; doi:10.1186/1471-2105-10-228)

## Slide 1
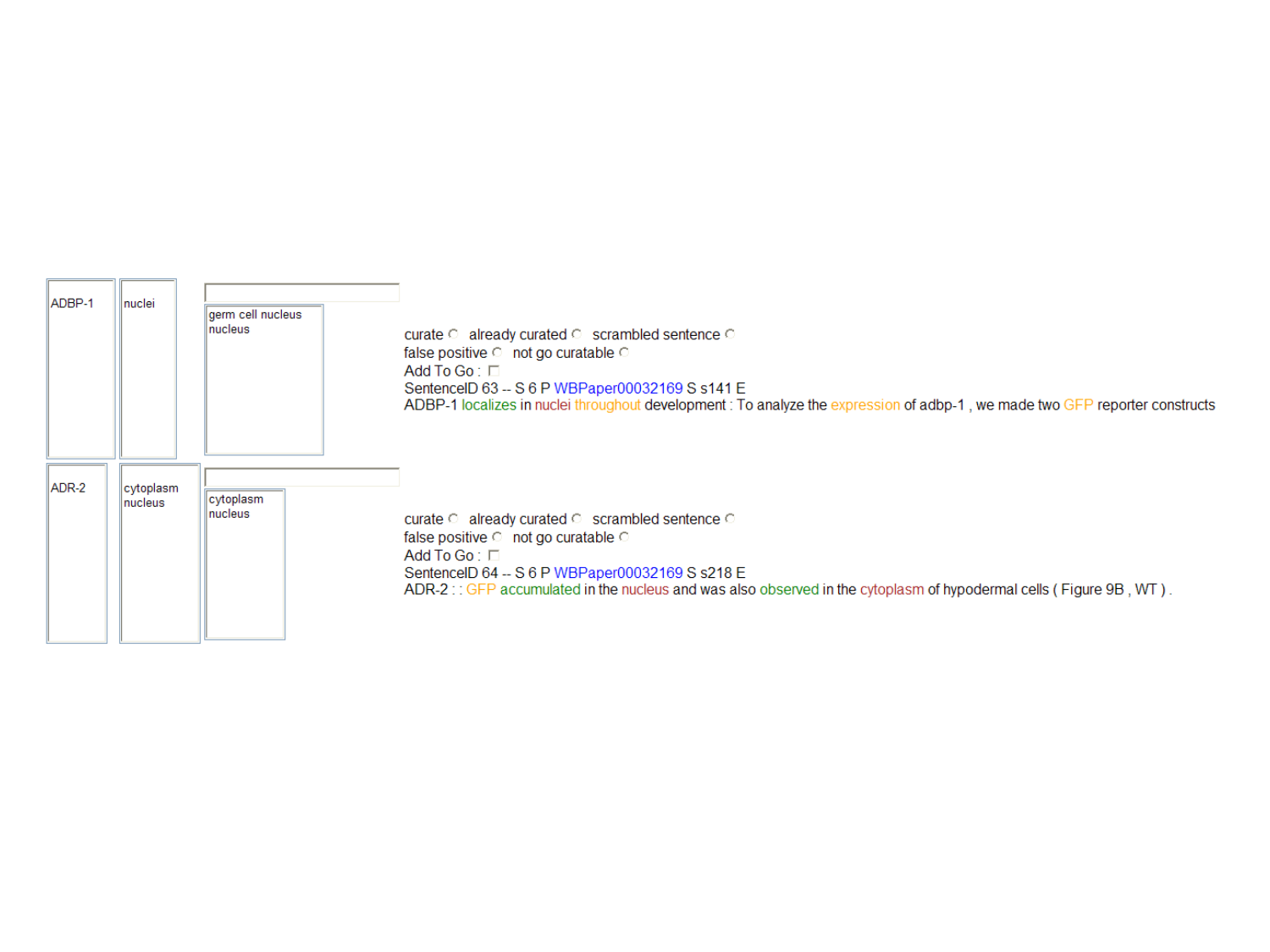

Supplement: Additional file 5 — WormBase Cellular Component curation form. This file shows a screenshot of the web-based curation form used for Textpresso-based GO curation at WormBase. The identified C. elegans protein is listed in the left-most box, with the component term(s) from the sentence listed in the middle box. Suggested GO annotations, based upon previous curation, are listed in the right-most box. The sentence from which the protein and component are derived are shown on the right, along with the possible actions that can be taken by a curator, including curating the information (and adding to GO), marking the information as already curated, or marking the returned sentence as 'scrambled', false positive, or not GO curatable for additional reasons, e.g. the sentence describes localization in a mutant background. [file 1471-2105-10-228-S5.ppt]
